# Estimating the burden of pediatric HIV in an ‘A’ category district in India: an epidemiological study

**DOI:** 10.1186/s12887-021-02836-4

**Published:** 2021-08-27

**Authors:** Anju Sinha, Reynold Washington, Rajeev Sethumadhavan, Rajaram Subramanian Potty, Shajy Isac, Vasantha Thavraj, Ravindra Mohan Pandey

**Affiliations:** 1 Division of Reproductive Biology, Maternal and Child Health, Indian Council of Medical Research, New Delhi, India; 2 St John’s Research Institute, Bangalore, India; 3 Karnataka Health Promotion Trust, Belgaum, India; 4 Karnataka Health Promotion Trust, Bangalore, India; 5 Department of Biostatistics, All India Institute of Medical Sciences, New Delhi, India

**Keywords:** Estimation, Pediatric HIV, Prevalence, Burden

## Abstract

**Background:**

India lacks epidemiological information on the disease burden of pediatric HIV. The National AIDS Control Program (NACP) estimates the numbers of HIV-positive children as a proportion of adult persons living with HIV. A third of HIV-positive children die before their first birthday and a half before they reach their second birthday. The early detection of HIV is crucial for the prevention of morbidities, growth delays, and death among HIV-positive children.

**Methods:**

The study aimed to estimate the disease burden of pediatric HIV among children in ‘A’ category district of a high HIV prevalence state. An ‘A’ category district is defined by the presence of > 1% HIV prevalence among the general population, as estimated by HIV Sentinel Surveillance. The study used an innovative three-pronged strategy combining cross-sectional and longitudinal methods. The overall burden of pediatric HIV was calculated as a product of cases detected multiplied by a net inflation factor, for each of three strategies.

**Results:**

The existing pool of HIV infection in the district is estimated to be 3266 (95% CI: 2621–4197) HIV positive children < 15 years of age, in a mid-year (2013) projected child population of about 1.4 million, thus giving an HIV prevalence of 0.23% (CI: 0.19–0.30) among children (0–14 years of age). The proportion of children among all people living with HIV in the district works out to 10.4% (CI: 8.6–13.5%).

**Conclusions:**

The study estimate of 0.23% HIV prevalence among children (0–14 years of age) is higher than the NACP estimates (0.02) and is 2.5 higher than the Karnataka state estimate (0.09)^22^. Similarly, the proportion of children among all persons living with HIV in Belgaum district is 10.4% in this study, as against 6.54% for India. The study methodology is replicable for other settings and other diseases.

## Introduction

Globally, the number of persons living with HIV is on the rise and is projected to be an emerging threat to public health. Countries are not on track for the UNAIDS 90–90-90 targets by 2020 and SDG 95–95-95 by 2030 [1]. The targets indicate the proportion of persons living with HIV who will know their HIV status, are on anti-retroviral treatment, and are virally suppressed. The 2020 estimate of children living with HIV was 1.94 million [2]. There are few estimates of the magnitude of pediatric HIV. Mathematical modeling-based estimates using the Spectrum and Estimation and Projection Package (EPP) model include only the 15–45 age group within the population [3]. In India, a country with 1.3 billion people, children up to 18 years constitute 41% of the total population [4]. However, India lacks accurate estimates of the pediatric HIV burden. The National AIDS Control Program (NACP) provides estimates of pediatric HIV that are based on its proportion to adult infections. The NACP uses data available from other countries to arrive at this estimate [5]. The WHO recommended various methods to estimate pediatric HIV including case reporting, household surveys, immunization clinic surveys, in-school and out-of-school surveys, mortality data, and vital registration [6]. These methods have been used in countries with a high HIV prevalence: South Africa, Nigeria, Kenya, Thailand, Argentina, Mozambique, Malawi, Indonesia, and others [7–9]. Similarly, the WHO and UNAIDS recommended measurement of HIV prevalence among children (0–14 years) in settings where the HIV prevalence among women in the reproductive age is 5% or greater, with high fertility rates, low coverage of prevention of mother to child transmission (PMTCT) services and where resources to conduct a large sample survey among children is feasible [10, 11]. In India, HIV prevalence among women is low and resources for large sample surveys are limited.

The National Family Health Surveys provide estimates of India’s adult HIV prevalence but do not include HIV prevalence estimates among children, because of the large sample size that is required [12].

One of the goals of India’s National Strategic Plan to end AIDS (2017–2024) is the elimination of mother-to-child transmission (EMTCT) of HIV. Mother-to-child transmission (MTCT) remains the main cause of HIV infection among children in India. Despite the availability of tools and methods to prevent, identify and treat HIV in children, India’s performance on this front is sub-optimal. By 2020, 95% of pregnant women should have received testing for HIV and syphilis and 95% of estimated HIV-positive pregnant women should have been on antiretroviral treatment (ART) to achieve an MTCT of less than 5%. However, by 2015, India had registered only 74.2% of 29.7 million estimated pregnancies and only 59% of registered pregnant women were tested at least once for HIV, during the antenatal period. Only 45% of an estimated 35,255 HIV-positive pregnant women received ARV. Out of 10,677 live births among HIV-positive mothers, 85% of babies were tested at least once in 2016–17, but only 59% were tested within 2 months, by which time many babies could have died. The lack of technology-enabled platforms and inadequate utilization of front-line workers for this purpose results in a linkage loss at every level [13]. The ‘start free, stay free and AIDS free’ platform offers India an opportunity for focused, coordinated action and renewed commitments to end Pediatric AIDS and to eliminate MTCT [14]. In the absence of ART, approximately 30% of untreated HIV-positive children die before their first birthday and more than 50% die before they reach 2 years of age [1]. Early diagnosis and timely initiation of treatment, substantially improves survival^2^. Untreated HIV in children results in growth delays that may not be reversed by ART [15, 16]. It is, therefore, crucial to have reliable estimates of Pediatric HIV, to plan, implement and monitor the coverage of prevention and care programs for children living with HIV.

The Indian Council of Medical Research commissioned a task force study to estimate the burden of Pediatric HIV in a category ‘A’ district of a high prevalence state. The study protocol has been previously published [17]. The NACP defines ‘A’ category district as one with an HIV prevalence > 1% among the general population, as estimated by HIV Sentinel Surveillance. This paper describes the results of the task force study.

## Methods

### Study design

We followed up a cohort of HIV-positive pregnant women to derive HIV incidence among children exposed to maternal HIV. In addition, cross-sectional analyses were used to estimate HIV prevalence among children (0–14 years) living within families with an index person living with HIV and among children attending outpatient departments from a stratified random sample of public and private health care facilities in the district.

### Study setting

Information regarding study setting, design, and sample size estimation have been previously described^17^. District Belgaum, with an HIV prevalence of 1.43% in 2011, was selected for the study from among three high prevalence districts in south India. In 2011, Belgaum district had a total population of 4,779,661 (Males: 50.7%, Females: 49.3%); 75% were rural residents and 73.5% literate (Males: 82.2%; Females: 64.6%) [18]. The district Belgaum has 10 sub-district divisions referred to as blocks or talukas. The coverage of antenatal care, including HIV testing as prevention of mother to child transmission (PMTCT) services was high (> 85%) and the district administration was supportive of the study.

All 971 government and private health care facilities (HCFs) were mapped [19]. We extracted HIV testing data from all 285 HCFs located within ten talukas with stand-alone HIV testing facilities (149 Govt. and 136 private) for the study. The study team visited the identified HCF twice every week for the duration of the study.

In Strategy 1 we used a prospective cohort design to measure the incidence rate of HIV among infants and young children (0–24 months) born to HIV-positive pregnant women. A crude line-list of HIV-positive pregnant women, prepared from the secondary data collected from the HCFs, was refined by removing duplicates and those ineligible (resident outside the district, died, currently neither pregnant nor recently delivered a baby). All HIV-positive pregnant women who consented to age-specific HIV blood tests for their infants were enrolled in the study. Trained field investigators contacted the women over the phone and visited them at home or other venues preferred by the woman. The visits were scheduled once a month during pregnancy and until the baby was 24 months old. Age-appropriate HIV testing in infants using DNA PCR dry blood spot (DBS) was conducted at 6–10 weeks and 6–9 months. Antibody-based ELISA tests were conducted at 18–24 months. Field investigators filled data on a mother and child form. Field supervisors validated the data before it entered into the study database.

In Strategies II and III we used a cross-sectional design. In Strategy II, using age-appropriate testing as described above, we examined HIV prevalence among children (0–14 years) residing in a family with an index person living with HIV. We compiled the list of index persons living with HIV from Integrated Counseling and Testing Centers (ICTC), blood banks, and community-based NGOs in all 10 talukas. The taluka is a sub-district unit, also referred to as a block. We de-duplicated the line-list and organized individuals into family units after field investigators validated this information during a visit to the home of the index person living with HIV. Any person living with HIV, of age 18–49 years, having a biological child aged 0–14 years residing in any taluka of Belgaum, who consented for HIV testing of their spouse and children were considered as eligible and included in strategy II of the study. Field investigators recorded demographic details and HIV testing data on a Family form.

In Strategy III, over a period of 4 months, we screened sick children visiting 10 selected out-patient health care facilities. We selected 10 health care facilities using random sampling, stratified by government and non-government sector, and by primary, secondary, or tertiary levels of health care from four of the 10 talukas. Indian experts adopted the Integrated Management of Childhood Illnesses-HIV (IMCI-HIV) criteria that were applicable only to children 0–5 years of age, to include its application among children aged 5–14 years, into the algorithm. They also developed operational definitions for each criterion in the algorithm before training health care providers from the four participating talukas. Sick children (0–14 years) presenting with suspected signs and symptoms and satisfying the ‘Modified Integrated Algorithm’, were tested at health care facilities using age-appropriate HIV tests [20]. The research team visited these facilities once weekly to supervise the process and to compile and track HIV test results. HIV prevalence was calculated for ‘sick’ children and included a list of the newly detected HIV-positive children.

**Fig. 1:**
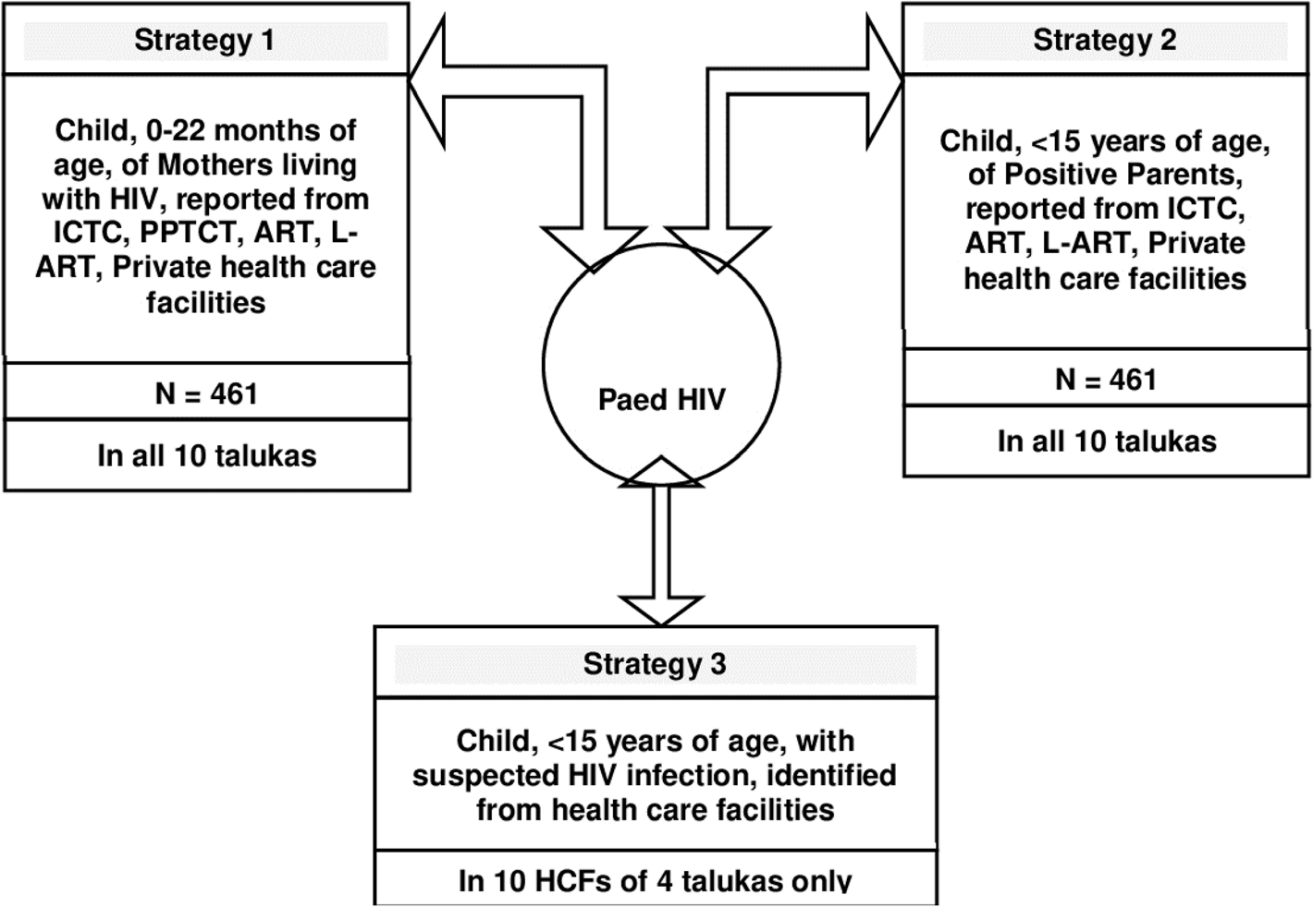
Outline of the research study

We derived estimates of children (0–14 years) living with HIV from each of the strategies (1, II & III) by applying inflation factors. The team of study investigators and the Project Advisory Group derived these inflation factors during a three-day workshop. The inflation factors took into consideration the population characteristics within the district, including total population (adult and child), estimated overall adult HIV prevalence, estimated prevalence among pregnant women, and reported coverage of HIV testing among the antenatal sub-population. The outline of the study design is depicted in Fig. 1.

The Institutional Ethics Committee of St. John’s Medical College, Bangalore provided ethics approval, and NACO, the Karnataka State AIDS Prevention Society (KSAPS), and District AIDS Prevention and Control Unit (DAPCU, Belgaum district NACO issued the regulatory approvals.

### Study implementation

We divided the ten talukas in the district into three clusters, each of which had five Field Investigators (FIs) and one supervisor (Senior Research Fellow, SRF). In each cluster, a team of one male and one female FI was given charge of two talukas for strategies 1 and 2. We appointed one additional FI for the strategy 3 related work. In total, we recruited and trained 14 FIs, over a duration was 10 days. The training was into two sessions that covered both technical information and soft skills for maintaining confidentiality and a gender-sensitive approach. SRFs planned daily visits of FIs validated 5% of data and verified forms for accuracy and completion. One medically qualified study coordinator supervised the entire field study.

### Data management & statistical analysis

Two data entry operators double entered all data using Microsoft Access. A statistician cleaned and verified the data for consistency and conducted the analyses using SPSS version 22.0 and STATA version 13.0.

A Project Advisory Group (PAG), constituted by national-level experts, guided the study team to develop the Statistical Analysis Plan (SAP). The primary outcome in Strategy 1 was cumulative incidence, calculated as the number of new infections per total number of children at risk. A child remained at risk till the first positive result by age-appropriate testing. For censored observations, time was the duration of follow-up. The SAP considered the limitations in coverage of services and response rates and guided the determination of the ‘Net inflation factor’ for each of the three strategies. Under Strategy I,the net inflation factor was derived using the estimated number of pregnancies in the district, proportion of un-tested pregnancies, pregnant mothers not enrolled, and un-tested children. In Strategies II and III, the prevalence of HIV infection among children 0–14 years of age was calculated. In alignment with the SAP, the actual/projected 18–49-year-old population in the study period, the estimated number of 0–14 year children, the proportion of eligible index persons not recruited, and the proportion of eligible children not tested were factors considered for the inflation process. In Strategy III, the factors considered for Net Inflation factor were: actual/projected 0–14 year population during the study period, the estimated number of 0–14-year-old children experiencing any morbidity, estimated morbid children reaching an HCF for care, estimated children satisfying the screening algorithm, geographical and institutional factors, morbid children not reaching selected HCF but reaching other facilities in the district, and proportions of children un-screened, non-enrolled and not HIV tested. The estimates derived under the strategies were multiplied by the inflation factor to come up with the overall estimate. The steps followed are described along with the results section.

## Results

In Strategy 1, we line listed 750 HIV-positive pregnant women from 285 selected health care facilities. After exclusion of duplicates, those non-resident or who had moved outside the district, died, or were no longer pregnant, we recruited 469 HIV-positive mothers who had delivered between 2011 and 2013, and who consented to participate in the cohort study. The total number of pregnancies among these 469 HIV-positive mothers was 496, as 27 mothers had a repeat pregnancy during the study period. The outcomes of these 496 pregnancies were 477 (96%) deliveries with at least one live birth, 10 (2%) abortions, and 9 (2%) stillbirths. Among the deliveries with live-births, 10 were twin pregnancies. Thus, the total number of live-born babies was 487. Of 487 HIV-exposed live-born children, 454 (92.3%) children were tested at least once, and 38 were found to be HIV positive during follow-up by 24 months of age. Only 400 of 487 babies (82%) received the first test between age 6–10 weeks; 23 died even before they reached the age for HIV testing. An additional 41 and 13 babies were tested by the second test and the third test was conducted between age 6–9 months and 18–24 months of age, thus bringing the total number of babies ever HIV tested to 454 (93%). 10 babies were completely lost to follow-up and remained never tested. The net cumulative incidence rates of vertical transmission of HIV per 100 pregnancies were 2.1 (95% CI: 1.1–3.8), 5.3 (95% CI: 3.6–7.8), and 7.8 (95% CI: 5.7–10.7) at 0–10 weeks, 0–9 months and 0–24 months of age, respectively. The cumulative incidence rate by 24 months was calculated to be 4.8% ((95% CI: 3.5–6.7).

**Fig. 2:**
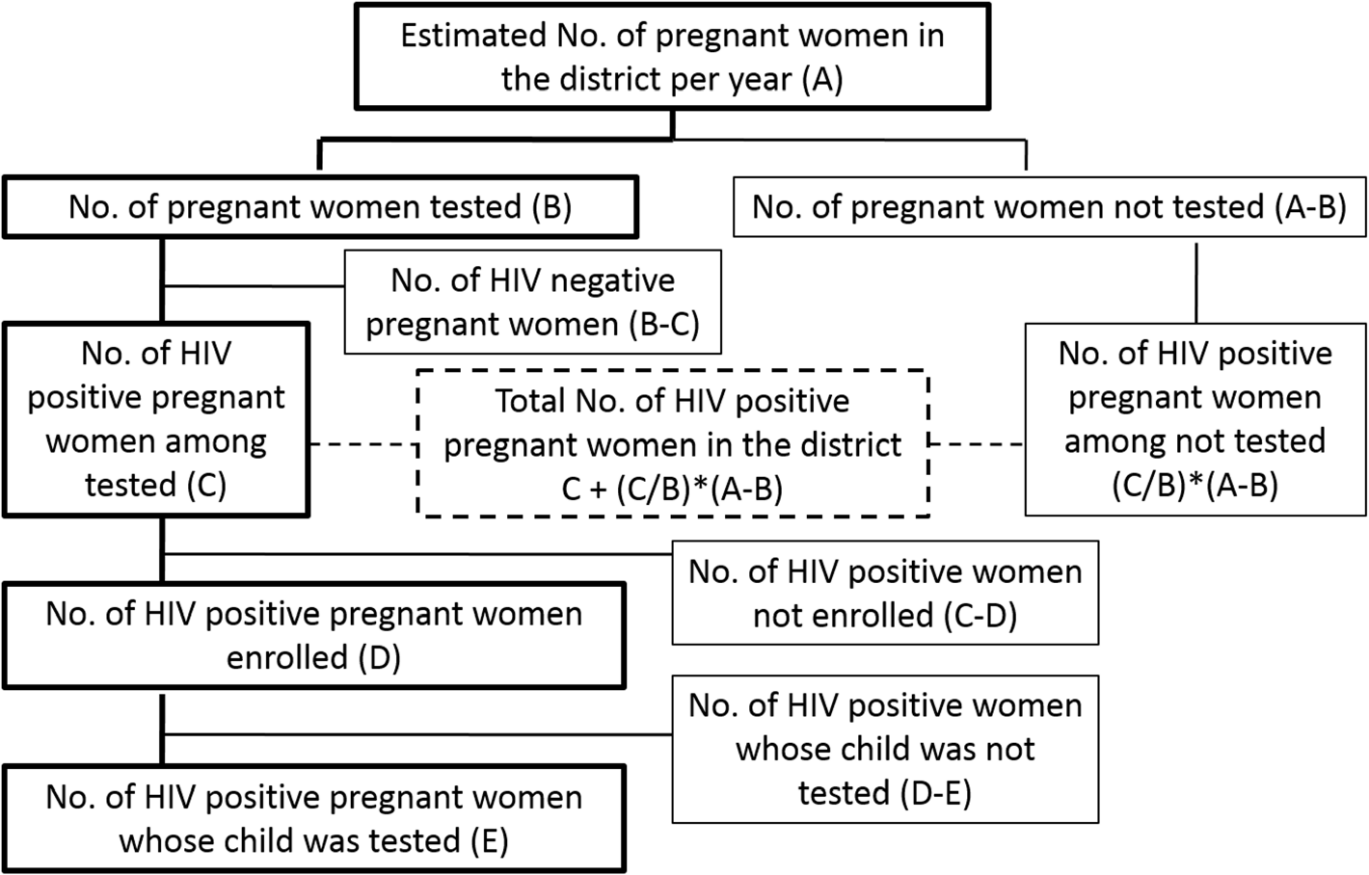
Conceptual diagram for calculating inflation factors and burden of Pediatric HIV from Strategy 1

To calculate the inflation factor at a population level, the following indicators as per the flow diagram (Fig. 2) were conceptualized. The basic parameters were taken from the Census data (Table 1). Based on this, the indicators in the flow diagram were deduced as in Table 2. Based on this inflation factor, the occurrence of new Pediatric HIV infections from MTCT is 41.2 (rounded off to 41) children by the age of 24 months.

**Table 1:** Census data for Belgaum district, 2011, considered for calculating inflation factors and burden of Pediatric HIV, Strategy 1

Sl. No.	Parameter	Total	Male	Female
1	Population, Belgaum district, 2001	4,207,264	2,147,746	2,059,518
2	Population, Belgaum district, 2011	4,779,661	2,423,063	2,356,598
3	Annual Growth Rate	0.012756	0.012061	0.013475
4	Projected population, Belgaum district, April 2012 (Mid-study year population)	4,841,019	2,452,465	2,388,567
5	The proportion of 15–49 years population, 2011	0.534365	0.536863	0.531796
6	Crude Birth Rate, Belgaum district, 2011 (Source: CRS, 2011)	19.71		
7	Estimated pregnancies, Belgaum district, 2012	95,416

**Table 2:** Calculation of inflation factors for Strategy 1

Sl. No.	Indicators in the Flow chart for Strategy 1	Number
8	No. of pregnant women tested (Source: PPTCT data, Belgaum district, 2012)	90,070
9	No. of pregnant women not tested (=(7)–(8))	5346
10	No. of HIV positive pregnant women among tested (Source: PPTCT data, Belgaum district, 2012)	187
11	HIV positivity among pregnant women (=(10)/(8))	0.002076
12	No. of HIV positive pregnant women among those not tested (=(11)*(9))	11
13	Total no. of HIV positive pregnant women in the district, 2012 (=(10) + (12))	198
14	Total no. of HIV positive pregnant women in the district during the study period (29 months)	479
15	No. of HIV positive mother-child pairs enrolled in the study	487
16	No. of enrolled HIV pregnant women whose child was tested	454
17	Inflation factor (=[(15)/(16)]*[(14)/(15)])	1.05

^a^Footnote: 469 HIV infected pregnant women were recruited from 469 Households (HH). More-than-one-time pregnancies of the same woman during study period = 27. Hence total HIV infected pregnancies = 469 + 27 = 496. Abortion = 10; Stillbirth = 9.Twins = 10.Total Mother-Child pairs = 496 + 10 = 506. Total live births = 506–19 = 487

**Fig. 3:**
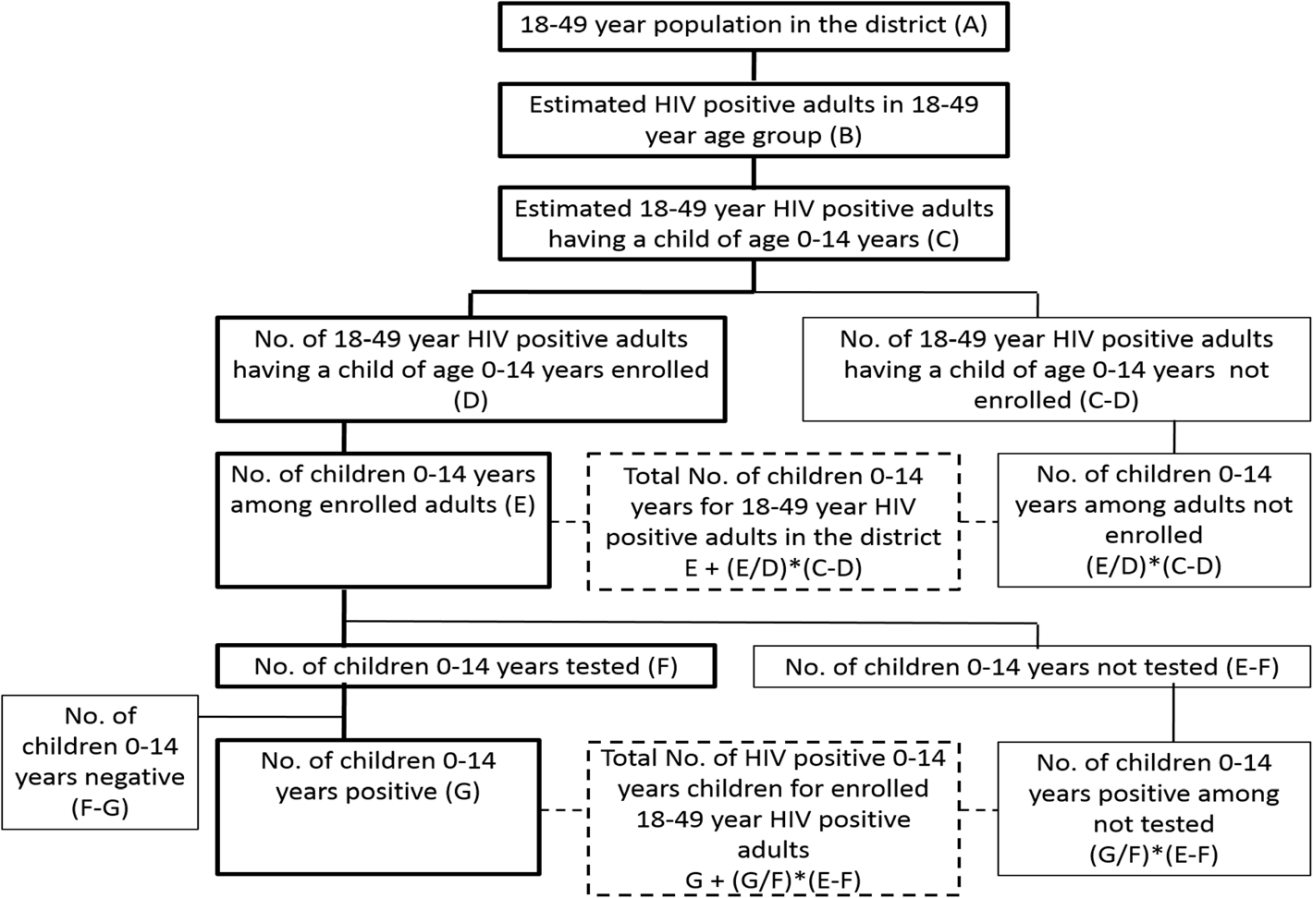
Conceptual diagram for calculating inflation factors and burden of Pediatric HIV from Strategy 2

In Strategy 2, we recruited 563 households (with 1062 children (Male:565; Female 497) from 671 eligible for recruitment, after eliminating duplication, including those not residents, migrated from the district or could not be contacted. 206 households from Strategy 1, who had children from previous pregnancies, were also included in this sample. Thus, from the 769 Households with 1388 children of age < 15 years, 1241 (90.1%) were tested for HIV; 131 tested HIV positive, giving an HIV prevalence of 10.6% ((95% CI: 8.6–13.5%). (Males: 12.8%; Females: 8.4%). The HIV prevalence was 18.6% (M: 23%, F:13%) in the < 5 year age group and 8% (M:9.2, F:6.9%) in the 5–14 years old age group. In order to project these findings to the district population, we used the criteria shown in Fig. 3.

The basic parameters that were required were taken from the Census data available (Table 3). We adopted the calculation shown in (Table 4) for estimating PLHIV in the district of Belgaum. We deduced the inflation factor as shown in the flow diagram in (Table 5).

**Table 3:** Census data for Belgaum district, 2011, considered for calculating inflation factors and burden of Pediatric HIV, Strategy 1 & 2

Sl. No.	Parameter	Total
1	Projected population, Belgaum district, Feb 2012 (Mid study population) (From Table 19 above)	4,841,019
2	The proportion of 15–49 years population, Belgaum district, 2011	0.534365
3	The proportion of 18–49 years population, 2011	0.478696
4	Projected 15–49 years population, Belgaum district, Feb 2012	2,586,871
5	Projected adult 18–49 years population, Belgaum district, Feb 2012	2,317,376
6	No. of children of age 0–14 years, Belgaum district, 2011	1,366,381
7	Projected children population (0–14 years), Belgaum district, Feb 2012	1,383,922
8	Child/Adult ratio, Belgaum district, Feb 2012	0.597193

**Table 4:** PLHIV estimation^a^, Belgaum district

Sl. No.	Parameter		Number
9	Sex ratio (Source: Census data, 2011)		960
10	Percentage of men having high-risk sexual activity		6.0
11	Estimated FSWs		11,766
12	Estimated MSM T		2333
13	Estimated IDUs		37
14	Estimated clients (=(1)*[1000/(1000 + (9))]*((10)/100)		79,190
15	Total high risk population (=(11) + (12) + (13) + (14))		93,326
16	Remaining 15–49 years population (=(7)–(15))		2,493,545
17	HIV prevalence, FSW	Min	16.40
18		Max	27.34
19	HIV prevalence, MSM	Min	5.51
20		Max	10.62
21	HIV prevalence, IDU	Min	2
22		Max	2
23	HIV prevalence, Clients	Min	3.85
24		Max	8.55
25	HIV prevalence, ANC	Min	0.69
26		Max	0.95
27	Adjusted HIV prevalence, ANC	Min	0.69
28		Max	0.95
29	Number of HIV positive FSWs	Min (=(11)*(17)/100)	1930
30		Max (=(11)*(18)/100)	3217
31	Number of HIV positive MSM	Min (=(12)*(19)/100)	129
32		Max (=(12)*(20)/100)	248
33	Number of HIV positive IDUs	Min (=(13)*(21)/100)	1
34		Max (=(13)*(22)/100)	1
35	Number of HIV positive Clients	Min (=(14)*(23)/100)	3049
36		Max (=(14)*(24)/100)	6771
37	Number of HIV positive general population	Min (=(16)*(27)/100)	17,205
38		Max (=(16)*(28)/100)	23,689
39	Total Number of HIV positive in the district	Min (=(29) + (31) + (33) + (35) + (37))	22,313
40		Max (=(30) + (32) + (34) + (36) + (38))	33,925
41	Average Total PLHIV 18–49 years, Belgaum district (=(39) + (40)/2)		28,119

^a^Sl. Nos. 10–13, 17–26: Source: Estimation of HRGs, Technical Report, NACO, 2009

**Table 5:** Calculation of inflation factors for Strategy 1 & 2

Sl. No.	Indicators in the Flow chart for Household information analysis	Number
42	Estimated 18–49 year HIV positive adult having a child of age 0–14 years (=(41)*(8)).	16,792
43	No. of 18–49 year HIV positive adult having a child of age 0–14 years enrolled	769
44	No. of 18–49 year HIV positive adult having a child of age 0–14 years not enrolled (=(42)–(43)).	16,023
45	No. of children 0–14 years of age for enrolled adults.	1388
46	Average no. of children per enrolled adult (=(45)/(43)).	1.805
47	Expecting same proportion of children for adults not enrolled, No. of children 0–14 years of age for adults not enrolled (=(46)*(44)).	28,921
48	Total No. of children 0–14 years of age for 18–49 year HIV positive adult in the district (=(45) + (47)).	30,309
49	No. of children 0–14 years of age (for enrolled adults) tested in the study.	1241
50	No. of children 0–14 years of age (for enrolled adults) not tested (=(45)–(49))	147
51	No. of children 0–14 years of age for enrolled adults tested positive in the study	131
52	HIV positivity among the tested children (=(51)/(49)).	0.10556
53	Expecting same proportion of HIV positivity among children not tested, No. of HIV positive 0–14 years children among untested children (=(52)*(50)).	16
54	Total No. of 0–14 years HIV positive children for enrolled 18–49 year HIV positive adult (=(51) + (53)).	147
55	Estimated children (0–14 years) HIV positive in the district (=(48)*(52)).	3199
56	At 95% CI of the prevalence to the estimate	2594–4094

In Strategy 3, of the total 33,342 children who visited the 10 health care facilities during the study period, 24,342 (73%) were screened by the trained field investigators. 527 (2.2%) sick children were identified, 509 completed HIV testing requirements. Of these, 97 children turned out to be positive (HIV prevalence 19.1%), but 86 of them had prior knowledge of their HIV-positive status. The study was, therefore, able to identify 11 (2.16%) new HIV-positive children from among the total of 509 sick children. For Strategy 3, it was assumed that HIV infection would be nil among the 23,815 screened children who did not have any indicative symptoms or social risk criteria for HIV and AIDS. The parameters considered are given in (Table 6).

**Table 6:** Calculation of inflation factors for Strategy 3

Sl. No.	Parameter	Total
1	Population, Belgaum district, 2001	4,207,264
2	Population, Belgaum district, 2011	4,779,661
3	Annual Growth Rate	0.012756
4	Projected population, Belgaum district, 2014	4,966,110
5	The proportion of 0–14 years population, Belgaum district, 2014	0.285874
6	Estimated 0–14 year population, Belgaum district, 2014	1,419,682
7	Assuming 10% of children as morbid, estimated no. of children experiencing any morbidity in the district in a year	141,968
8	Assuming 70% of these morbidities/clinic visits are unique children, expected no. of unique morbid children in the district in a year (=(7)*0.7	99,378
9	During the study period (127 days), a total no. of children reached at HCFs for any morbidity	33,342
10	During the study period (127 days), no. of children screened at HCFs (127 days)	24,342
11	Assuming 70% of these morbidities/clinic visits are unique children, expected no. of unique morbid children in the district in a year (=(10)*0.7	17,039
12	No. of children not screened (even if they reached at HCF in 127 days + had the screening been done for the remaining 238 days in a year) (=(8)–(10))	82,339
13	No. of children screened positive (sick children) and enrolled in the study	527
14	No. of unique children screened positive (sick children) and enrolled in the study	515
15	No. of enrolled sick children tested for HIV in the study	509
16	No. of unique enrolled sick children tested for HIV in the study	497
17	No. of tested sick children found positive in the study	97
18	No. of unique tested sick children found positive in the study	89
19	No. of unique newly detected unique HIV positive children among (18)	11
20	Percent of new unique positive children identified among all unique screened positive (sick children) (=(19)/(16))	0.022
21	If all children were tested, new positive children that could be identified from the study (=(14)*(20))	11.40
22	Estimated unique children who would have been identified as sick among the unscreened (=[(14)/(11)]*(12))	2488.7
23	Estimated no. of unique positive children among the unscreened (=(20)*(22))	55.1
24	Total new unique positive children that in the district in a year (=(23) + (21))	67
25	At 95% Confidence Interval of prevalence to the estimate	27–103

### Estimating the burden of HIV in children < 15 years

**Fig. 4:**
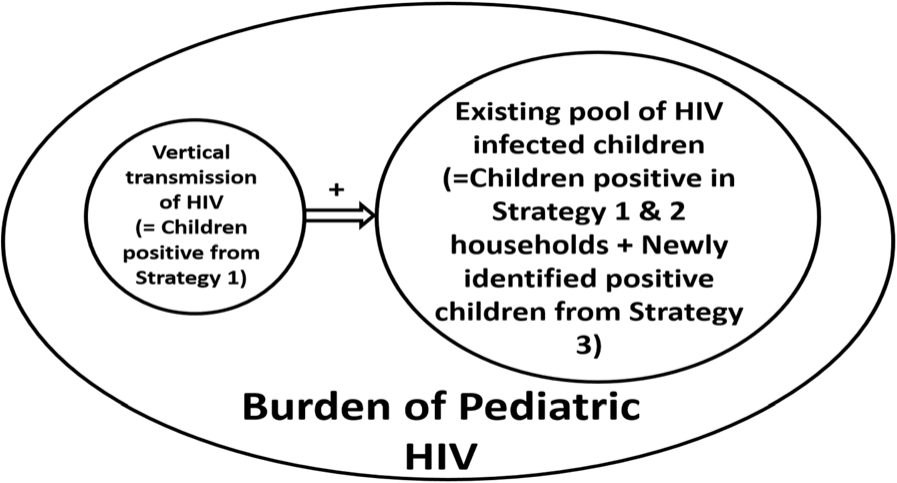
Conceptualization of calculating the burden of HIV among Children

We estimated the burden of children 0–14 years by considering all three strategies as shown in (Fig. 4) Data obtained through the cross-sectional approach in the study is indicative of the existing pool of HIV infection among children in the district for the point in time. Assuming other methods of transmission are nil, the vertical transmission of HIV (Strategy I) adds to the existing pool. Mortality among HIV-positive children decreases the existing pool. The total number of children in the existing pool of HIV infection in Belgaum district in the study is the sum of children HIV positive in Strategy I and II, and newly identified children from Strategy III, this is given in Table 7.

**Table 7:** Calculation of existing pool of Pediatric (< 15 years) HIV infection

Strategy	HIV Prevalence (%)	HIV pool of infection
1 & 2	10.5 (95% CI: 8.6–13.5)	3199 (95% CI: 2594–4094)
3	2.2 ^a^(95% CI: 0.9–3.4)	67 (95% CI: 27–103)
Total	–	3266 (95% CI:2621–4197)

^a^ additional HIV detected from sick children, not previously identified in families

Thus, the existing pool of HIV infection in the district works out to be 3266 (95% CI: 2621–4197) HIV positive children < 15 years of age, in a mid-year (2013) projected child population of about 1.4 million, thus giving us an overall HIV prevalence of 0.23% (95% CI: 0.19–0.30) among children (0–14 years of age). The proportion of children among all people living with HIV in the district works out to 10.4% (95% CI: 8.6–13.5%).

Assuming that other methods of transmission of HIV to children < 15 years of age group is nil, the net cumulative incidence rate (%) of vertical transmission of HIV per 100 pregnancies is 4.8% (95% CI: 3.5–6.7%) by about 24 months of child’s age.

## Discussion

This is the first district-wide study estimating the Pediatric HIV burden in India. The findings indicate that Belgaum district had an overall HIV prevalence of 0.23% among children (0–14 years) and about 3266 children living with HIV, during the year 2013, making the proportion of Pediatric infections 10.4% of all HIV infections in the district. This is about a third higher than the national estimates (based on projections of adult infections) ranging between 6 and 7% of all HIV infections, during the same period [21, 22]. The results of this study could be generalized to other similar districts with high HIV prevalence, high coverage of HIV screening during pregnancy/delivery, and high levels of treatment coverage among those living with HIV. The results although not generalizable across all districts in India, do provide useful information on how to estimate the burden of Pediatric HIV from existing data by using an excel based software (being published elsewhere).

The GBD framework based on Spectrum and EPP uses multiple methodological improvements, yet faces limitations and biases due to the use of variable data sources. A non-parametric back-calculation method used in Thailand studied HIV/AIDS trends for future predictions. This study reported the use of data adjustments to overcome surveillance reporting issues. Moreover, these methods did not include pediatric age groups [3, 23]. This study is an effort to derive the pediatric burden estimate from existing data within a district.

The uniqueness of this study is its innovative epidemiological design, using a robust combination of community and facility-level data, the inclusion of private and public sector health facilities, the multi-pronged strategy of using cross-sectional and longitudinal data collection techniques, and the extrapolation to correct for gaps in coverage using inflation factors. In most countries, including India, linkage loss tends to occur at various levels within the PMTCT programs [24]. The study staff supported the program to reduce the gaps in HIV testing and treatment linkages at various levels. They thus achieved high levels of coverage of testing of children identified within the families of index persons living with HIV, as well as timely testing of the HIV exposed newly delivered infants. With well-defined line listing and recruitment processes in place and individualized follow-up, the study was able to quantify and reduce duplication and to better understand the mobility of PLHIV within and outside the district. A number of doctors were also trained in the use of the Modified Integrated Algorithm based on IMCI-HIV guidelines, during the study. The response rates of eligible subjects for HIV testing were high with about 85% of eligible mothers completing the protocol for follow-up and a similar proportion of eligible families with children completing HIV testing for all children. The study methodology is resource-intensive but may be replicable for other settings and other diseases.

However, we also recognize some limitations in the study. During the study period, many changes in the policy of HIV treatment and prophylaxis for pregnant women occurred including prophylaxis using single-dose Nevirapine, the use of expanded regimens to option B, option B+, and a ‘test and treat all HIV positive pregnant women, irrespective of CD4 count, at the time of HIV diagnosis. HIV-related prevention and treatment services were already district-wide and to scale. As a result of prevention and treatment initiatives, there were steady declines in HIV prevalence among the general population, and particularly among pregnant women that led to a delay in completion of the required sample size and follow up in Strategy I. Our results showed that 51% of all pregnant women had been initiated on Anti-retroviral treatment (ART), 42% during pregnancy, and 9% after delivery. The effect of the mother’s HIV treatment on the incidence of HIV among exposed children has been published [25]. The reduced incidence of HIV among pregnant women delayed achievement of the required sample size for strategy 1, which could have implications in our calculation of prevalence when using the mid-year estimated population size for the district. However, because of the large overall population size within the Belgaum district, the effect of these annual changes in incidence on estimating the size of children living with HIV, may not be substantial.

A second limitation is the assumption of a similar prevalence among the non-included subjects within the study, during the extrapolation exercise. The number of variables that we had collected was insufficient to completely match the characteristics of responders with non-responders. The non-response bias could make our estimate higher or lower than the true figure. However, with high levels of coverage as achieved in the study, it is unlikely to change the overall estimate.

There was a delay in initiation of Strategy III, due to delays in the development of the Modified Integrated Algorithm by the national experts/ICMR sub-committee, and the training of health care providers in selected talukas. A large majority (86 of the 97; 89%) of HIV-positive children detected by this strategy had prior knowledge of their HIV-positive status, indicative of the wide coverage of HIV testing among children in the district. However, despite this, **Strategy III** did yield new HIV positives among children attending a health facility for reasons other than HIV treatment, thus suggesting one more modality of closing the gap in HIV testing among children who have been exposed to maternal HIV during years when timely age-appropriate HIV testing services were not available or accessible.

Our study only considered children within a family unit. We did not include children who lived without a parent (child-headed homes) or who lived within an institution. Previous studies and Strategy 3 results indicate that children who were orphans were much more likely to be HIV positive. We did pick up some of the HIV-positive orphans in Strategy 3. However, our sampling design and small numbers did not allow us to estimate the HIV prevalence among orphans. A cross-sectional HIV prevalence survey of children living in orphanages could have added value to this study.

Another primary assumption used in strategy I was that the new cases were contributed only by mother-to-child transmission. Recent studies from other countries have indicated that adolescents orphaned as a result of HIV are at greater risk and vulnerability for physical and sexual abuse, including HIV [26]. However, it is expected that these other modes of HIV transmission are rare and the numbers that add to the burden would be minimal. Lastly, we could not integrate mortality and migration estimates into this estimation. The study was not designed to systematically measure mortality among children living with HIV and the information on age-specific mortality rates for children 0–14 years were not available. We could not calculate age-specific death dates from the current study, as reporting of data for child deaths in the family was not forthcoming and the records were unavailable for verification of the actual date of death, even when they were reported. We observed that HIV prevalence among the 0–5 year age group was more than twice the HIV prevalence in the children 5–14 years. Interventions for PMTCT or treatment of HIV and AIDS in Belgaum were almost non-existent 10 years before the study period. Therefore, the only plausible reason for this reduction in prevalence in a cross-sectional strategy II could be that most children living with HIV had died. The non-integration of mortality information into the estimate could have resulted in a higher than the true value.

Despite these limitations, the study is the first of its kind in India and offers new information on methods to estimate Pediatric HIV. We put forward several recommendations for further studies. During the phase II ongoing study, it would also be useful to explore the feasibility of testing the baby for HIV at birth [27], as many maternally exposed new-born died before they were due for the first HIV test at 6 weeks. Testing for HIV at birth is a current recommended CDC guideline that has not yet been adopted in India [28]. Prolonged breastfeeding beyond 6 months, directly increases the risk of HIV infection, especially when it is not exclusive [25]. The acquisition of HIV infection among the children of age group post 24 months need to be further explored by ensuring follow-up HIV testing of the child at least 4–6 weeks following cessation of breastfeeding. A cohort study of all children within sampled family units impacted by HIV would also provide insights into mortality and morbidity among non-infected and HIV positive children, into their nutritional health status, and the influence of the health status of the mothers living with HIV [29]. It is acknowledged that there could be other reasons for HIV transmission to children, especially amongst adolescents [30]. A cohort study among adolescents within these families could indicate the extent to which this occurs.

## Conclusions

The study has used a unique innovative methodology for disease burden estimation of pediatric HIV in a high prevalence district in India, where such data do not exist. Our study estimate of the proportion of pediatric HIV among all PLHIV is 10.4%, higher than the earlier projected figure of 6%. The study methodology, though resource-intensive, can be replicated in similar settings for HIV as well as for other infectious diseases.

## Data Availability

The datasets generated and/or analyzed during the current study are not publicly available. The study data is available only to the collaborating scientists. The data may be available on request to the corresponding author Dr. Anju Sinha (apradhandr@gmail.com), Indian Council of Medical Research (ICMR), New Delhi. The data may also be available upon request for some of the collaborating institutions. Data will be sanitized to remove individual identifiers in order to comply with the local data protection laws. All data sharing is subject to National AIDS Control Organisation (NACO) and ICMR approval.

## Abbreviations

HIV: Human immunodeficiency virus
AIDS: Acquired immune deficiency syndrome
NACP: National AIDS Control Program
WHO: The World Health Organization
UNAIDS: The United Nations Program on HIV and AIDS
EMTCT: Elimination of Mother-to-Child Transmission of HIV and syphilis
ICTC: Integrated counselling and testing centers
MTCT: Mother-to-child transmission
PPTCT: Prevention of parent-to-child transmission
PMTCT: Prevention of mother to child transmission
ART: Anti-retroviral therapy
DNA-PCR: Deoxyribonucleic acid -polymerase chain reaction
IMCI-HIV: Integrated Management of Childhood Illness- human immunodeficiency virus
IMAI: Integrated Management of Adolescent and Adult Illness (IMAI)
KSAPS: Karnataka State AIDS Prevention Society
DAPCU: District AIDS Prevention and Control Unit
PAG: Project Advisory Group
SAP: Statistical Analytical Plan
PLHIV: Persons living with HIV
FSW: Female sex worker
MSM: Men who have sex with men
IDU: Injecting drug users
EPP: Estimation and Projection Package
CDC: Centers for Disease Control
CD4: Cluster of differentiation 4
